# Assessment of lung injury severity using ultrasound in critically ill COVID-19 patients in resource limited settings

**DOI:** 10.1186/s13613-023-01133-w

**Published:** 2023-04-27

**Authors:** Seydina Alioune Beye, Boubacar Diallo, Modibo Keita, Yacouba Cissoko, Khadidia Ouattara, Hammadoun Dicko, Majaliwa Shabani, Amadou Sidibé, Modibo Berthé, Yaya Ibrahim Coulibaly, Nouhoum Diani, Mohamed Keita, Yacouba Toloba, Sounkalo Dao, Veronique Suttels, Youssouf Coulibaly, Armand Mekontso Dessap

**Affiliations:** 1 Department of Intensive Care and Anesthesia, Point G Teaching Hospital, Bamako, Mali; 2 Faculty of Medicine and Odonto-Stomatology (FMOS)/University of Sciences, Technics and Technologies of Bamako (USTTB), Bamako, Mali; 3 Department of Public Health, Teaching Hospital (CHU) Dermatology, Bamako, Mali; 4 Department of Infectious Diseases and Tropical Diseases, Point G Teaching Hospital, Bamako, Mali; 5 Department of Pneumology, Point G Teaching Hospital, Bamako, Mali; 6 Department of Intensive Care and Anesthesia/University of Sciences, Technics and Technologies of Bamako (USTTB), Bamako, Mali; 7 National Teaching Hospital for Tuberculosis and Respiratory Diseases (CNHU-PPC), Cotonou, Benin; 8 Service de Médecine Intensive Réanimation, AP-HP, Hôpitaux Universitaires Henri-Mondor, 94010 Créteil, France; 9 Univ Paris Est Créteil, CARMAS, 94010 Créteil, France; 10 Univ Paris Est Créteil, INSERM, IMRB, 94010 Créteil, France

**Keywords:** Lung ultrasound, Assessment, COVID-19 patients, ICU, Mali

## Abstract

**Background:**

Lung ultrasound is a non-invasive tool available at the bedside for the assessment of critically ill patients. The objective of this study was to evaluate the usefulness of lung ultrasound in assessing the severity of SARS-CoV-2 infection in critically-ill patients in a low-income setting.

**Methods:**

We conducted a 12-month observational study in a university hospital intensive care unit (ICU) in Mali, on patients admitted for COVID-19 as diagnosed by a positive polymerase chain reaction for SARS-CoV-2 and/or typical lung computed tomography scan findings.

**Results:**

The inclusion criteria was met by 156 patients with a median age of 59 years. Almost all patients (96%) had respiratory failure at admission and many needed respiratory support (121/156, 78%). The feasibility of lung ultrasound was very good, with 1802/1872 (96%) quadrants assessed. The reproducibility was good with an intra-class correlation coefficient of elementary patterns of 0.74 (95% CI 0.65, 0.82) and a coefficient of repeatability of lung ultrasound score < 3 for an overall score of 24. Confluent B lines were the most common lesions found in patients (155/156). The overall mean ultrasound score was 23 ± 5.4, and was significantly correlated with oxygen saturation (Pearson correlation coefficient of − 0.38, p < 0.001). More than half of the patients died (86/156, 55.1%). The factors associated with mortality, as shown by multivariable analysis, were: the patients’ age; number of organ failures; therapeutic anticoagulation, and lung ultrasound score.

**Conclusion:**

Lung ultrasound was feasible and contributed to characterize lung injury in critically-ill COVID-19 patients in a low income setting. Lung ultrasound score was associated with oxygenation impairment and mortality.

**Supplementary Information:**

The online version contains supplementary material available at 10.1186/s13613-023-01133-w.

## Background

According to the World Health Organization regional office for Africa (WHO AFRO), 8.9 million cases of COVID-19 and 174,000 deaths have already been reported in Africa, which represent < 5% of the global burden [1]. However, seroprevalence and modelling studies indicate that the number of COVID-19 cases in the African region is similar to that of other WHO regions. This discrepancy may illustrate the lack of resources in terms of adequate SARS-CoV-2 detection strategies which should largely rely on microbiological or immunological tests [2].

The clinical presentation of SARS-CoV-2 infection ranges from mild respiratory symptoms to severe pneumonia progressing to diffuse alveolar damage leading to hypoxemic acute respiratory failure [3]. The estimated prevalence of acute respiratory failure in affected patients is between 15 and 20% [4, 5], and the percentage of admission to intensive care unit (ICU) among hospitalized patients varies from 4 to 47% [6].

Prevalence data from low- and middle-income countries (LMIC) on SARS-CoV-2 acute respiratory distress syndrome (ARDS) are limited. In African critical care settings, 30-day in-hospital mortality reaches almost 50%, which is significantly higher than their Asian, European, or American counterparts. This high mortality is associated with insufficient critical care resources, comorbidities such as HIV/AIDS, and severe organ dysfunction at admission [7].

Radiologically, COVID-19 pneumonia is seen as bilateral pulmonary infiltrates. In less severe cases, computed tomography (CT-scan) shows bilateral ground-glass opacities, predominantly subpleural (45–62% of cases) [8], and areas of sub-segmental consolidation, whereas in more severe cases, CT-scan shows lobar and sub-segmental consolidation [9]. Lung ultrasound is an established diagnostic tool widely used in the management of dyspnea in emergency and critical care units in high income countries. The sensitivity and specificity of lung ultrasound for the detection of pneumonia are superior to those of standard chest X rays (CXR) and are close to those of CT-scan [10] for other forms of lung injury [11]. For such, lung ultrasound is now recommended as an alternative to CXR for the diagnosis of pneumonia. With its new low cost portable devices, point-of-care lung ultrasound is an attractive tool in LMIC settings. However, there is not enough information on its use in constrained environments [12]. During the COVID-19 pandemic in Mali, access to CT-scan was difficult since patients were improperly cohorted and healthcare personnel were at high risk of exposure to the virus due to shortage of personal protective equipment outside dedicated COVID-19 units. The situation was worsened by the urgent need for oxygen at admission. Therefore, we hypothesized that bedside lung ultrasound would be a feasible interesting alternative to appraise the clinical severity in order to guide patient management. This study assessed the feasibility of lung ultrasound in the evaluation of the extent of lung injury in severe COVID-19 infection in a resource-limited setting.

## Methods

This was a 12-month observational study conducted in a university hospital in Mali from March 2020 to February 2021. The study recruited patients of any age and sex with a confirmed diagnosis of COVID- 19 infection based on a positive Polymerase Chain Reaction test for SARS-CoV-2 and/or typical CT scan findings. Patients were included upon admission to the intensive care unit (ICU), which was fully dedicated to critically-ill COVID-19 patients) and were subjected to pleuro-pulmonary ultrasound at admission. We excluded patients with chronic respiratory diseases.

### Lung ultrasound

An ultrasound device (VINNO, V5) equipped with a high-frequency linear and a low-frequency convex probes was used. The standardized procedure of lung ultrasound was performed in the semi-recumbent position, as previously described, with twelve areas analyzed (Additional file 1: Table S1). Briefly, we used the convex probe depending on the patient's body type, with a unifocal mode centered on the pleural line, we avoided saturation phenomena (by reducing the gain and the mechanical index), and avoided all filters and other image acquisition modalities to achieve the highest possible frequency [13].

In case the patient was unable to sit up, the operator would look for a partial view of the posterior side of the chest as that area is considered as hot spots in COVID-19 infection. If this was not possible, the lung ultrasound imaging would start from the paravertebral lower quadrant area above the diaphragm.

**Fig. 1:**
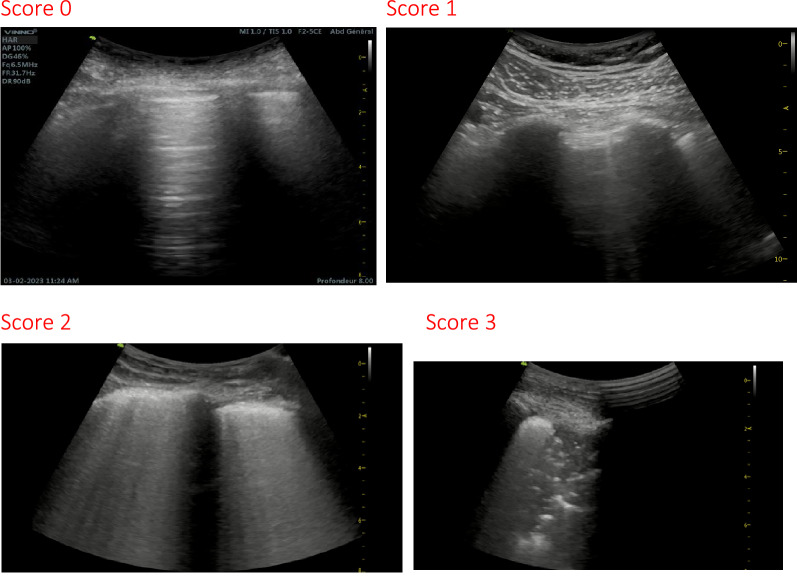
Examples of elementary scores

The calculation of the lung ultrasound score was based on the elementary score of each quadrant, where the latter ranges from 0 to 3, as previously proposed [13, 14]: 0 if the pleural line was continuous and regular, with presence of horizontal artifacts (A lines); 1 in case the pleural line showed sawtooth appearance (irregular), with vertical lines (comet tails) visible below the pleural line; 2 if the pleural line was interrupted, with small areas of consolidation visible below the points of interruption of the pleural line; and 3 in case of large extensive areas of “white lung” with or without large areas of pulmonary consolidation (see examples of elementary scores in Fig. 1). The global lung ultrasound score ranged from 0 to 36 (add up of the scores of 12 quadrants). We also recorded the following features seen on lung ultrasound: presence of A line, irregular pleural line or B line, white lung, and areas of pulmonary consolidation.

### Data collection

The following parameters were collected: age, sex, comorbidities (hypertension, diabetes, obesity, chronic obstructive pulmonary disease, heart failure), room air transcutaneous oxygen saturation (SpO_2_), acute respiratory distress syndrome (ARDS, as per Berlin criteria), organ failures (as per sequential organ failure assessment, SOFA), lung ultrasound score and features, ventilatory support, and outcome (hospital death and length of stay).

### Assessment of lung ultrasound reproducibility

Fifteen recordings (from fifteen separate patients) were selected from the study to assess reproducibility. The same sets of recordings were analysed separately by two different ultrasonographers to assess inter-analyser reproducibility.

### Data analysis

Data were analyzed with R software version 3.6.1. The dependent variable was death presented in dichotomy (yes and no). Explanatory variables were grouped into patients’ clinical and biological characteristics, ultrasound parameters, and management. Descriptive analysis of all variables was performed with relative frequencies for categorical variables and with median, and interquartile range for quantitative variables. For the bi-variate analysis, Mann Whitney, Chi-square, and Fisher's exact tests were used as appropriate. Given the number of events, a reasonable number of eight explanatory variables that had a p-value of less than 0.05 in the bivariate analysis were retained for the multivariable analysis. A binomial logistic regression was conducted with the step-down method, and the likelihood test was used to compare the different intermediate models. The lowest Akaike criterion (AIC) was considered to retain the final model. The adequacy of the final model and the intermediate models was verified using Hosmer and Lemeshow test [15]. Models and their ROC (Receiver Operating Characteristic) curve areas were compared using the Likelihood ratio test. The reproducibility of lung ultrasound elementary patterns is expressed by the intra-class correlation coefficient [25], determined with consistency and 95% confidence interval. The reproducibility of lung ultrasound score is expressed by the coefficient of repeatability [26], as proposed by Bland and Altman. Coefficient of repeatability is calculated as the British Standards Institution repeatability coefficient (twice the standard deviation of the differences in repeated measurements) [26].

## Results

### Patients

Over the study period, 156 patients meeting our criteria were included (Additional file 1: Table S2). The overall mean age was 59.27 ± 18.06 and the male/female sex ratio was 1.73 (99/57). Comorbidities were present in 67.9% (106/156) of the patients. Almost all patients had respiratory failure (150/156) at admission while one third and one fifth had hemodynamic failure (48/156) and renal failure (35/156), respectively (Additional file 1: Table S2)**.**

### Lung ultrasound findings

**Fig. 2:**
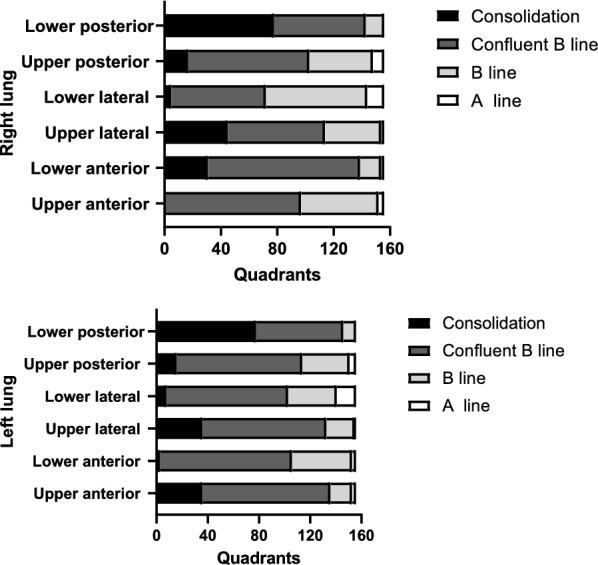
Findings of lung ultrasound in different lung quadrants

**Fig. 3:**
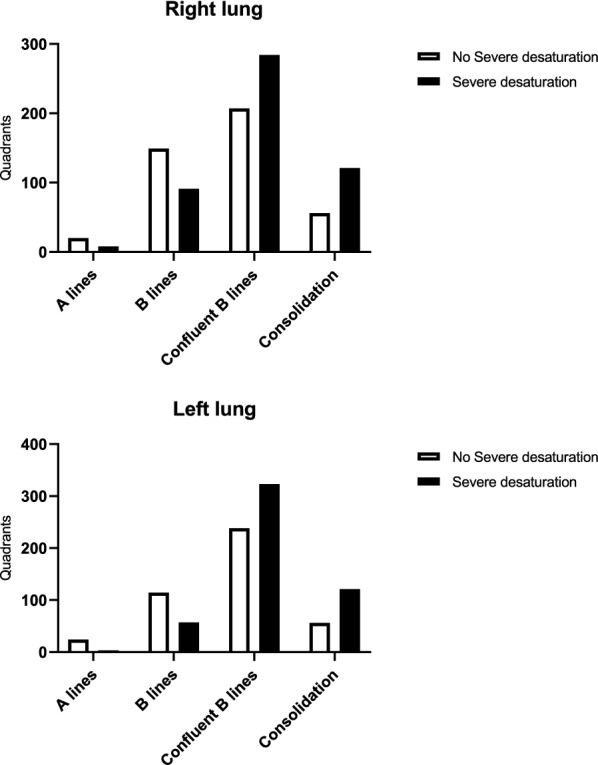
Findings of lung ultrasound in patients presenting with severe desaturation and those without

Feasibility of lung ultrasound was 1802/1872 of the assessed quadrants. Lung lesions were present bilaterally in all assessed patients. The description of lung ultrasound features in different lung quadrants is depicted in Fig. 2. Overall, confluent B lines were the most common findings (155/156), followed by few B lines (116/156) (Additional file 1: Table S3). The overall mean ultrasound score was 23 ± 5.4. There was a significant negative correlation between the ultrasound score and pulsed oxygen saturation (Pearson correlation coefficient of − 0.38, p < 0.001). Patients presenting with profound desaturation (as defined by a pulsed oxygen saturation below 85% at ICU admission) had a lower lung ultrasound score as compared to their counterparts (20.7 ± 5.4 vs 25.0 ± 5.4, p < 0.001). Patients presenting with severe desaturation also had more confluent B lines and consolidations than their counterparts (p < 0.001, Fig. 3).

### Reproducibility of lung ultrasound

The intra-class correlation coefficient of lung ultrasound elementary patterns was 0.74 (95% CI 0.65, 0.82). The coefficient of repeatability of lung ultrasound score was 2.12, for an overall score of 24.

### Management

The vast majority of patients received dexamethasone-based corticosteroid therapy (138/150, 92%), with antibiotic therapy (143/151, 94.7%) often driven by nosocomial bacterial infections (63/143, 44%). Many patients needed respiratory support (121/156, 77.56%) either non-invasive (75/121, 62%) or invasive (46/121, 38%). For the management of severe ARDS, 48/142 (33.8%) were placed in prone position after tracheal intubation. Only 8.6% (13/151) patients received prophylactic heparin therapy, while the majority (138, 91.39%) received therapeutic anticoagulation (Additional file 1: Table S2)**.**

### Outcome

More than half of the patients died (86/156, 55.1%). Deceased patients were older and more often frail, or hypertensive. On the other hand, asthma, sickle cell disease, and pregnancy were more prevalent in survivors (Additional file 1: Table S2). Deceased patients had more organ failure during the ICU course than did the survivors (Additional file 1: Table S2). Mean ultrasound score was significantly higher in deceased than in surviving patients (Additional file 1: Table S3 and Figure S1). Confluent B lines and consolidations were more common in non-survivors as compared with survivors (Additional file 1: Table S3).

The multivariable analysis showed that the factors associated with mortality were patients’ age, number of organ failures, therapeutic anticoagulation, and lung ultrasound score (Additional file 1: Table S4). The model predicting mortality had a larger area under the ROC curve with integration of lung ultrasound than without (p = 0.002, Figure S2).

## Discussion

The unprecedented burden of the 2019 coronavirus pandemic (COVID-19) has required healthcare facilities to implement robust and easy-to-use strategies to prioritize management and predict outcomes [13]. In this study, we explored the value of the lung ultrasound score in critically-ill COVID 19 patients in a LMIC setting. Lung ultrasound was a perfectly feasible investigation tool, and a higher lung ultrasound score was correlated with oxygenation impairment and associated with mortality as evidenced in the multivariable analysis. In a multicenter cohort study conducted in ten African countries, the mortality of patients with COVID 19 appeared to be higher than it was in developed countries. Their multivariable analysis demonstrated that such higher mortality was associated with insufficient human resources in ICUs, presence of comorbidities (HIV/AIDS, diabetes, chronic liver disease, and renal disease), and number of organ failures at the time of ICU admission [16].

Pneumonia appears to be the most severe manifestation of SARS-CoV-2 infection [6, 10]. The peripheral distribution of pulmonary infiltrates in COVID-19 makes ultrasound a reliable imaging tool that can reduce the number of follow-up CT-scans [3, 10, 17], the associated risks of infection spread and radiation exposure. In addition, transporting critically ill patients is difficult and complex, whereas ultrasound can be easily performed at the bedside. Lung ultrasound is a diagnostic tool with proven efficiency in identifying severe forms and their evolution [18–20]. During this COVID-19 pandemic, lung ultrasound was used sporadically in several centers to assess disease severity and to help guide treatment decisions [3, 13].

Our data show that higher lung ultrasound scores were significantly associated with hypoxemia and clinical severity. The mechanisms of hypoxemia during COVID-19 pneumonia are debated, with some authors reporting “silent hypoxemia”[21] or suggesting that hypoxemia is not only due to the extent of non-aerated tissue [22]. However, intracardiac (patent foramen ovale) or intrapulmonary (transpulmonary bubble transit) shunt do not seem to be the main drivers of hypoxemia in COVID-19 pneumonia [23]. Our results suggest a significant association between the degree of lung injury and that of hypoxemia during COVID-19 pneumonia. The pre-eminence of confluent B-lines over consolidation is in line with studies suggesting that the major component of the venous admixture in COVID-19-ARDS is ventilation-perfusion mismatch (i.e., perfusion of poorly ventilated lung regions), rather than true right-to-left shunt (i.e., perfusion of non-aerated tissue) [22]. Our results are also in accordance with previous studies suggesting that an increase in lung ultrasound score is associated with in-hospital mortality [24, 25]. Lung lesions of COVID-19 lead to intrapulmonary shunt [26] and to dysregulation of pulmonary perfusion [27]. Our findings prove that lung ultrasound is a simple tool to assess severity and to predict outcomes [17, 28, 29]. The high sensitivity and specificity of lung ultrasound [30–32] make it an important tool for the detection of severe forms.

The intra-class correlation coefficient value for lung ultrasound elementary pattern was close to 0.75, indicating a substantial agreement between the two raters [33, 34]. The intra-class correlation coefficient is the proportion of the total variance which is due to the variation between the subjects (a value of 1 indicates that the total variance is due solely to the variation between the subjects while a value of 0 indicates that the total variance is attributed to variation between observers) [35]. We also found a good reliability of lung ultrasound score assessment, with a coefficient of repeatability close to 2, for an overall score of 24. The coefficient of repeatability is the smallest significant difference between repeated measurements [36]. In our study, this means that an absolute change of 2 or more in lung ultrasound score may therefore be required for accurate interpretation if the recordings are analysed by different observers in the setting tested. The good reproducibility of lung ultrasound in this study is in accordance with findings in high income settings [37].

In our work, advanced age and organ failures were associated with death, which is consistent with the results of previous reports [38]. Similarly, our finding on anticoagulation is in line with previous studies suggesting that therapeutic anticoagulation does not improve the survival of patients with severe COVID-19 admitted to ICU, and may increase the risk of bleeding in unselected patients [39, 40].

One strength of this prospective study is its focus on critically-ill patients in a low resources context in Sub-Saharan Africa. Some teams use a single holistic microconvex probe for all bedside ultrasound (neither linear nor abdominal nor cardiac), a strategy that may make ultrasound even more affordable in constrained environments [41]. The main limitations of this study are its monocentric nature and the lack of biological parameters of patients.

## Conclusion

In conclusion, our study suggests that lung ultrasound could serve as a valuable tool for the detection and prognostication of lung injury in critically-ill patients with COVID-19 pneumonia in resource limited settings. This simple, bedside accessible, and reliable tool has a particular potential in resource limited settings in such situations. Larger multicenter studies are needed to validate our findings.

## Supplementary Information

**Additional file 1: Table S1.** Description of the quadrants considered for ultrasound examination. **Table S2.** Sociodemographic characteristics, clinical presentation and management during intensive care unit stay. **Table S3.** Lung ultrasound findings in survivors and nonsurvivors. **Table S4.** Factors associated with death by multivariable logistic binomial regression. **Figure S1**. Findings of lung ultrasound in survivors and deceased patients. **Figure S2.** Receiver operating characteristics curves for prediction of hospital death incorporating or not lung ultrasound score.

## Data Availability

All data and methods supporting the findings of this study are available from the corresponding author upon reasonable request.
