# Supplementary material for: Assessment of lung injury severity using ultrasound in critically ill COVID-19 patients in resource limited settings
Source: Ann Intensive Care. 2023 Apr 27;13:33. doi: 10.1186/s13613-023-01133-w (PMC10134692; doi:10.1186/s13613-023-01133-w)
Supplement: Supplementary file 1 — Additional file 1: Table S1. Description of the quadrants considered for ultrasound examination. Table S2. Sociodemographic characteristics, clinical presentation and management during intensive care unit stay. Table S3. Lung ultrasound findings in survivors and nonsurvivors. Table S4. Factors associated with death by multivariable logistic binomial regression. Figure S1. Findings of lung ultrasound in survivors and deceased patients. Figure S2. Receiver operating characteristics curves for prediction of hospital death incorporating or not lung ultrasound score. [file 13613_2023_1133_MOESM1_ESM.docx]

# Supplementary Materials

## [Table S1: Description of the quadrants considered for ultrasound examination](file:///C:\Users\beyeseydinaalioune\Downloads\refwarticleecho\Table%20S1.docx)

|  | **Right side area** | **Quadrants** |
| --- | --- | --- |
| Back | 1. Low paravertebral above the diaphragm | Right lower posterior |
|  | 2. Middle paravertebral at the lower tip of the scapula | Right upper posterior |
| Axilla | 3. Middle axillary below the nipple line | Right lower lateral |
|  | 4. Middle axillary above the nipple line | Right upper lateral |
| Chest | 5. Middle clavicular below the nipple line | Right lower anterior |
|  | 6. Middle clavicular above the nipple line | Right upper anterior |
|  | **Left side area** | **Quadrant** |
| Back | 7. Low paravertebral above the diaphragm | Left lower posterior |
|  | 8. Middle paravertebral at the lower tip of the scapula | Left upper posterior |
| Axilla | 9. Middle axillary below the nipple line | Left lower lateral |
|  | 10. Middle axillary above the nipple line | Left upper lateral |
| Chest | 11. Middle clavicular below the nipple line | Left lower anterior |
|  | 12. Middle clavicular above the nipple line | Left upper anterior |

## Table S2: Sociodemographic characteristics, clinical presentation and management during intensive care unit stay

|  | | **All patients**  **N=156** | | | | | **Death** | | | | | | **p value** | |
| --- | --- | --- | --- | --- | --- | --- | --- | --- | --- | --- | --- | --- | --- | --- |
|  |  |  |  |  |  |  | **No** | | | **Yes** | | |  |  |
| **Variables** | |  |  |  |  |  | **n=70** | | | **n=86** | | |  |  |
| Age (years) | |  | | | | |  | | |  | | |  | |
| *Mean* | | 59.2±18.0 | | | | | 51.7±18.1 | | | 65.4±17.7 | | | <0.001 | |
| *1^st^ Quartile* | | 45.7 | | | | |  | | |  | | |  | |
| *Median* | | 62.5 | | | | |  | | |  | | |  | |
| *3^rd^ Quartile* | | 72.0 | | | | |  | | |  | | |  | |
| Sex | |  | | | | |  | | |  | | |  | |
| *Male* | | 99 (63.4%) | | | | | 45 (64.3%) | | | 54 (62.8%) | | | 0.86 | |
| *Female* | | 57 (36.5%) | | | | | 25 (35.7%) | | | 32 (37.2%) | | |  |  |
|  | |  | | | | |  | | |  | | |  | |
| Frailty scale | | N=120 | | | | | n=61 | | | n=59 | | |  | |
| *good condition* | | 68 (56.6%) | | | | | 32 (52.4%) | | | 36 (61.0%) | | | <0.001 | |
| *Weak* | | 18 (15.0%) | | | | | 3 (4.9%) | | | 15(25.4%) | | |  | |
| *very good condition* | | 32 (26.6%) | | | | | 26 (42.6%) | | | 6 (10.1%) | | |  | |
| *very weak* | | 2 (1.6%) | | | | | 0 (0.0%) | | | 2 (3.3%) | | |  | |
|  | |  | | | | |  | | |  | | |  | |
| **Past medical history** | |  | | | | |  | | |  | | |  | |
| Asthma | | 7 (4.4%) | | | | | 6 (8.6%) | | | 1(1.2%) | | | 0.04 | |
| Sickle cell disease | | 4 (2.5%) | | | | | 4 (5.7%) | | | 0 (0 %) | | | 0.03 | |
| High blood pressure | | 62 (39.7%) | | | | | 18 (25.7%) | | | 44 (51.3%) | | | 0.001 | |
| Diabetes Mellitus | | 29 (18.5%) | | | | | 12 (17.1%) | | | 17 (19.8%) | | | 0.83 | |
| Smoking | | 49 (31.4%) | | | | | 17 (24.3%) | | | 32 (37.2%) | | | 0.11 | |
| COPD | | 8 (5.1%) | | | | | 5 (7.1%) | | | 3 (3.5%) | | | 0.47 | |
| Pregnancy | | 18 (11.5%) | | | | | 13 (72.2%) | | | 5 (27.7%) | | | 0.004 | |
|  | |  | | | | |  | | |  | | |  | |
| **Organ failures** | |  | | | | |  | | |  | | |  | |
| Neurological failure | | |  | |  | | |  | | |  | | | |
| *No* | | | 126 (80.7%) | | 64 (91.4%) | | | 62 (72.1%) | | | 0.002 | | | |
| *Yes* | | | 30 (19.2%) | | 6 (8.6%) | | | 24 (27.9%) | | |  |  |  |  |
|  | | |  | |  | | |  | | |  | | | |
| Glasgow come Scale at ICU admission | | |  | |  | | |  | | |  | | | |
| *Mean* | | | 13.9 | | 14.7 | | | 13.2 | | | 0.001 | | | |
| *1^st^ Quartile,* | | | 15 | |  | | |  | | |  | | | |
| *Median* | | | 15 | |  | | |  | | |  | | | |
| *3^rd^ Quartile* | | | 15 | |  | | |  | | |  | | | |
|  | | |  | |  | | |  | | |  | | | |
| Respiratory failure | | |  | |  | | |  | | |  | | | |
| *No* | | | 6 (3.8%) | | 5 (7.1%) | | | 1 (1.2%) | | | 0.09 | | | |
| *Yes* | | | 150 (96.1%) | | 65 (92.9%) | | | 85 (98.8%) | | |  |  |  |  |
|  | | |  | |  | | |  | | |  | | | |
| SpO_2_ at ICU admission | | |  | |  | | |  | | |  | | | |
| *Mean* | | | 70.7 | | 87.0 | | | 73.8 | | | 0.001 | | | |
| *1^st^ Quartile,* | | | 71 | |  | | |  | | |  | | | |
| *Median* | | | 84 | |  | | |  | | |  | | | |
| *3^rd^ Quartile* | | | 90 | |  | | |  | | |  | | | |
|  | | |  | |  | | |  | | |  | | | |
| Acute Respiratory Distress Syndrome (ARDS*)* | | |  | |  | | |  | | |  | | | |
| *No* | | | 22 (14.1%) | | 17 (60.4%) | | | 5 (39.5%) | | | 0.001 | | | |
| *Yes* | | | 134 (85.9%) | | 53 (39.5%) | | | 81 (60.4%) | | |  |  |  |  |
| Hemodynamic failure | | |  | |  | | |  | | |  | | | |
| *No* | | | 108 (69.2%) | | 62 (88.6%) | | | 46 (53.5%) | | | 0.001 | | | |
| *Yes* | | | 48 (30.7%) | | 8 (11.4%) | | | 40 (46.5%) | | |  |  |  |  |
| Liver failure | | |  | |  | | |  | | |  | | | |
| *No* | | | 150 (96.1%) | | 70 (100%) | | | 80 (93.0%) | | | 0.03 | | | |
| *Yes* | | | 6 (3.8%) | | 0 (0%) | | | 6 (7.0%) | | |  |  |  |  |
| Kidney failure | | |  | |  | | |  | | |  | | | |
| *No* | | | 121 (77.5%) | | 63 (90%) | | | 58 (67.4%) | | | 0.001 | | | |
| *Yes* | | | 35 (22.4%) | | 7 (10%) | | | 28 (32.6%) | | |  |  |  |  |
| Number of organ failures | | |  | |  | | |  | | |  | | | |
| *Mean* | | | 1.7 | | 1.3 | | | 2.1 | | | 0.001* | | | |
| *1^st^ Quartile,* | | | 1 | |  | | |  | | |  |  |  |  |
| *Median* | | | 1 | |  | | |  | | |  |  |  |  |
| *3^rd^ Quartile* | | | 2 | |  | | |  | | |  |  |  |  |
|  | | |  | |  | | |  | | |  | | | |
| Sepsis | | | 89 (74.1%) | | 53 (75.7%) | | | 36 (41.9%) | | | 0.001 | | | |
| *No* | | | 67 (24.3%) | | 17 (24.3%) | | | 50 (58.1%) | | |  | | | |
| *Yes* | | |  | |  | | |  | | |  |  |  |  |
|  | | |  | |  | | |  | | |  |  |  |  |
| **Management** | | |  | |  | | |  | | |  |  |  |  |
| Corticosteroids | | | N=150 | | n=66 | | | n=84 | | |  | |  |  |
| *No* | | | 12 (8.0%) | | 5 (7.58%) | | | 7 (8.33%) | | | 0.56 | |  |  |
| *Yes* | | | 138 (92.0%) | | 61 (92.42%) | | | 77 (91.67%) | | |  |  |  |  |
|  | | |  | |  | | |  | | |  | |  |  |
| Antibiotics | | | N=151 | | n=66 | | | n=85 | | |  | |  |  |
| *No* | | | 8 (5.3%) | | 6 (9.09%) | | | 2 (2.35%) | | | 0.07 | |  |  |
| *Yes* | | | 143 (94.7%) | | 60 (90.91%) | | | 83 (97.65%) | | |  |  |  |  |
|  | | |  | |  | | |  | | |  | |  |  |
| Transfusion | | | N=150 | | n=66 | | | n=84 | | |  | |  |  |
| *No* | | | 133 (88.6%) | | 57 (86.3%) | | | 76 (90.4%) | | | 0.45 | |  |  |
| *Yes* | | | 17 (11.3%) | | 9 (13.6%) | | | 8 (9.5%) | | |  |  |  |  |
|  | | |  | |  | | |  | | |  | |  |  |
| Heparin | | | N=151 | | n=67 | | | n=84 | | |  | |  |  |
| *Therapeutic* | | | 138 (91.3%) | | 56 (83.5%) | | | 82 (97.6%) | | | 0.002 | |  |  |
| *Prophylactic* | | | 13 (8.6%) | | 11 (16.4%) | | | 2 (2.3%) | | |  |  |  |  |
|  | | |  | |  | | |  | | |  | |  |  |
| Catecholamine | | | N=156 | | n=70 | | | n=86 | | |  | |  |  |
| *No* | | | 100 (64.1%) | | 63 (90.0%) | | | 37 (43.0%) | | | 0.001 | |  |  |
| *Yes* | | | 56 (35.9%) | | 7 (10.0%) | | | 49 (57.0%) | | |  |  |  |  |
|  | | |  | |  | | |  | | |  | |  |  |
| Respiratory assistance | | | N=156 | | n=70 | | | n=86 | | |  | |  |  |
| *No* | | | 35 (22.4%) | | 26 (37.1%) | | | 9 (10.4%) | | | 0.001 | |  |  |
| *Yes* | | | 121 (77.5%) | | 44 (62.8%) | | | 77 (89.5%) | | |  |  |  |  |
|  | | |  | |  | | |  | | |  | |  |  |
| Prone position | | | N=142 | | n=64 | | | n=78 | | |  | |  |  |
| *No* | | | 94 (66.2%) | | 43 (67.1%) | | | 51 (65.3%) | | | 0.05 | |  |  |
| *Yes* | | | 48 (33.8%) | | 21 (32.8%) | | | 27 (34.6%) | | |  | |  |  |
|  | | |  | |  | | |  | | |  | |  |  |
| Hemodialysis | | | N=156 | | n=70 | | | n=86 | | |  | |  |  |
| *No* | | | 145 (92.9%) | | 66 (94.3%) | | | 79 (91.9%) | | | 0.08 | |  |  |
| *Yes* | | | 11 (7.0%) | | 4 (5.7%) | | | 7 (8.1%) | | |  | |  |  |

COPD, chronic obstructive pulmonary disease; SpO2, oxygen saturation, ICU: intensive care unit; p values are from Mann Whitney test or Pearson Chi2 test

## Table S3: Lung ultrasound findings in survivors and nonsurvivors

|  | **All patients N=156** | **Death** | | **p value** |
| --- | --- | --- | --- | --- |
|  |  | **No (n=70)** | **Yes (n=86)** |  |
| **Lung ultrasound score** | 23.0±5.4 | 21.04±5.41 | 24.60±5.39 | 0.001* |
| **Right lung** |  |  | |  |
| Right upper anterior quadrant |  |  |  |  |
| *Consolidation* | 1 (0.6%) | 0 (0.0%) | 1 (1.2%) | 0.001 |
| *A Line* | 4 (2.5%) | 3 (4.3%) | 1 (1.2%) |  |
| *B Line* | 55 (35.2%) | 34 (48.6%) | 21 (24.4%) |  |
| *Confluent B line* | 96 (61.5%) | 33 (47.1%) | 63 (73.3%) |  |
| Right lower anterior quadrant |  |  |  |  |
| *Consolidation* | 31 (19.8%) | 11(15.7%) | 20 (23.3%) | 0.006 |
| *A Line* | 2 (1.2%) | 2 (2.9%) | 0 (0.0%) |  |
| *B Line* | 15 (9.6%) | 12 (17.1%) | 3 (3.5%) |  |
| *Confluent B line* | 108 (69.2%) | 45 (64.3%) | 63 (73.3%) |  |
| Right upper lateral quadrant |  |  |  |  |
| *Consolidation* | 45 (28.8%) | 15 (21.4%) | 30 (34.9%) | 0.033 |
| *A Line* | 2 (1.2%) | 1 (1.4%) | 1 (1.2%) |  |
| *B Line* | 40 (25.6%) | 25 (35.7%) | 15 (17.4%) |  |
| *Confluent B line* | 69 (44.2%) | 29 (41.4%) | 40 (46.5%) |  |
| Right lower lateral quadrant |  |  |  |  |
| *Consolidation* | 5 (3.2%) | 1 (1.4%) | 4 (4.7%) | 0.006 |
| *A Line* | 12 (7.6%) | 8 (11.4%) | 4 (4.7%) |  |
| *B Line* | 72 (46.1%) | 40 (57.1%) | 32 (37.2%) |  |
| *Confluent B line* | 67 (42.9%) | 21 (30.0%) | 46 (53.5%) |  |
| Right upper posterior quadrants |  |  |  | 0.206 |
| *Consolidation* | 78 (50.0%) | 30 (42.9%) | 48 (55.8%) |  |
| *A Line* | 0 (0.0%) | 0 (0.0%) | 0 (0.0%) |  |
| *B Line* | 13 (8.3%) | 8 (11.4%) | 5 (5.8%) |  |
| *Confluent B line* | 65 (41.6%) | 32 (45.7%) | 33 (38.4%) |  |
| Right lower posterior quadrants |  |  |  |  |
| *Consolidation* | 78 (50.0%) | 30 (42.9%) | 48 (55.8%) | 0.206 |
| *A Line* | 0 (0.0%) | 0 (0.0%) | 0 (0.0%) |  |
| *B Line* | 13 (8.3%) | 8 (11.4%) | 5 (5.8%) |  |
| *Confluent B line* | 65 (41.6%) | 32 (45.7%) | 33 (38.4%) |  |
| **Left lung** |  |  |  |  |
| Left upper anterior quadrant |  |  |  |  |
| *Consolidation* | 3 (1.9%) | 0 (0.0%) | 3 (3.5%) | 0.002 |
| *A Line* | 3 (1.9%) | 2 (2.9%) | 1 (1.2%) |  |
| *B Line* | 47 (30.1%) | 30 (42.9%) | 17 (19.8%) |  |
| *Confluent B line* | 103 (66.0%) | 38 (54.3%) | 65 (75.6%) |  |
| Left lower anterior quadrant |  |  |  |  |
| *Consolidation* | 36 (23.0%) | 16 (22.9%) | 20 (23.3%) | 0.0906 |
| *A Line* | 3 (1.9%) | 2 (2.9%) | 1 (1.2%) |  |
| *B Line* | 17 (10.9%) | 12 (17.1%) | 5 (5.8%) |  |
| *Confluent B line* | 100 (64.1%) | 40 (57.1%) | 60 (69.8%) |  |
| Left upper lateral quadrant |  |  |  |  |
| *Consolidation* | 8 (8.1%) | 1 (1.4%) | 7 (8.1%) | 0.001 |
| *A Line* | 15 (9.6%) | 13 (18.6%) | 2 (2.3%) |  |
| *B Line* | 38 (24.3%) | 25 (35.7%) | 13 (15.1%) |  |
| *Confluent B line* | 95 (60.9%) | 31 (44.3%) | 64 (74.4%) |  |
| Left lower lateral quadrant |  |  |  |  |
| *Consolidation* | 36 (23.0%) | 14 (20.0%) | 22 (25.6%) | 0.014 |
| *A Line* | 1 (0.6%) | 1 (1.4%) | 0 (0.0%) |  |
| *B Line* | 22 (14.1%) | 16 (22.9%) | 6 (7.0%) |  |
| *Confluent B line* | 97 (62.1%) | 39 (55.7%) | 58 (67.4%) |  |
| Left upper posterior quadrant |  |  |  |  |
| *Consolidation* | 16 (10.1%) | 4 (5.7%) | 12 (14.0%) | 0.133 |
| *A Line* | 5 (3.2%) | 4 (5.7%) | 1 (1.2%) |  |
| *B Line* | 37 (23.7%) | 19 (27.1%) | 18 (20.9%) |  |
| *Confluent B line* | 98 (62.8%) | 43 (61.4%) | 55 (64.0%) |  |
| Left lower posterior quadrant |  |  |  |  |
| *Consolidation* | 78 (50.0%) | 37 (52.9%) | 41 (47.7%) | 0.028 |
| *A Line* | 0 (0.0%) | 0 (0.0%) | 0 (0.0%) |  |
| *B Line* | 10 (6.4%) | 8 (11.4%) | 2 (2.3%) |  |
| *Confluent B line* | 68 (43.5%) | 25 (35.7%) | 43 (50.0%) |  |

** Mann Whitney,*

## Table S4: Factors associated with death by multivariable logistic binomial regression

| **Factors** | **Univariate OR (95% CI)** | **Multivariable OR**  **(95% CI)** | | **LR-test** |
| --- | --- | --- | --- | --- |
|  |  | |  |  |
| Age, per year | 1.05 (1.03 – 1.07) | | 1.03 (1.01 – 1.06) | 0.006 |
| Number of organ failures | 3.44 (2.11 – 5.61) | | 3.56 (2.07 – 6.14) | < 0.001 |
| Lung ultrasound score | 1.15 (1.07 – 1.23) | | 1.13 (1.04 - 1.23) | 0.002 |
|  |  | |  |  |
| Anticoagulation |  | |  | 0.015 |
| Prophylactic (reference) | 1 | | 1 |  |
| Therapeutic | 7.83 (1.67 – 36.64) | | 8.2 (1.19 – 56.26) |  |

*Log-likelihood = -73.5*

*Number of observations = 156*

*AIC value = 157.1*

*Hosmer and Lemeshow test (binary model), p=0.96*

*CI denotes confidence interval*

## Figure S1: Findings of lung ultrasound in survivors and deceased patients

## Figure S2: Receiver operating characteristics curves for prediction of hospital death incorporating or not lung ultrasound score

| Without lung ultrasound score | With lung ultrasound score |
| --- | --- |
| 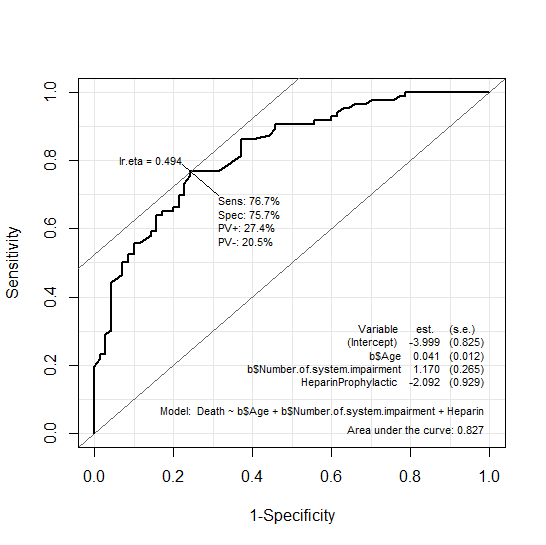 | 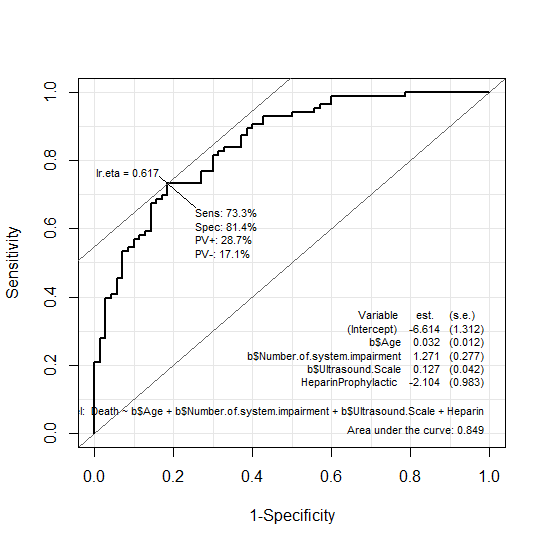 |
